# An Electrolytic Elemental Iron Powder Effectively Regenerates Hemoglobin in Anemic Rats and Is Relatively Well Absorbed When Compared to Ferrous Sulfate Monohydrate

**DOI:** 10.3390/nu16172833

**Published:** 2024-08-24

**Authors:** James H. Swain, Logan D. Glosser, Caroline J. Jang, Ryan C. Nemeth, Anshul R. Bethi, Eva L. Zheng, Evelyn R. Boron, Hannah M. Fox

**Affiliations:** 1 Department of Nutrition, School of Medicine, Case Western Reserve University, 10900 Euclid Avenue, Cleveland, OH 44106, USA; 2 School of Medicine, Emory University, 100 Woodruff Circle, Atlanta, GA 30322, USA; 3 Scientific Enrichment Opportunity Program, School of Medicine, Case Western Reserve University, 10900 Euclid Avenue, Cleveland, OH 44106, USA; 4 Baylor University Medical Center, 3500 Gaston Ave, Dallas, TX 75246, USA

**Keywords:** electrolytic elemental iron powder, hemoglobin regeneration efficiency, relative bioavailability, fortification

## Abstract

Elemental iron powders are used as food fortificants to reduce the incidence of iron deficiency anemia. However, many commercially available iron powders are relatively untested in vivo. The purpose of this study was to determine the hemoglobin regeneration efficiency (HRE) and relative iron bioavailability (RBV) of an electrolytic elemental iron powder (EIP), by treating anemic rats with 14 d iron repletion diets fortified with four different concentrations (12, 24, 36, or 48 mg iron/kg diet) of EIP and bakery-grade ferrous sulfate monohydrate (FS; FeSO_4_•H_2_O), or no added iron (control); n = 9–12/group. The HRE of FS was significantly higher (*p* ≤ 0.05) than EIP at each concentration of dietary iron tested. For EIP, the HREs (ratios) of diets containing 12, 24, 36, and 48 mg iron/kg were 0.356, 0.205, 0.197, and 0.163, respectively. For both EIP and FS, HRE was inversely associated with increasing dietary iron. The RBVs (%) of iron from EIP in diets at 12, 24, 36, and 48 mg iron/kg as compared to FS were 64.5, 59.1, 50.6, and 54.3%, respectively. Overall, findings show that at the concentrations of iron tested, EIP has RBVs greater than 50% and is an effective fortification agent to replenish hemoglobin and correct iron deficiency anemia.

## 1. Introduction

Processed foods are often enriched and fortified with different forms of elemental iron powders to reduce the incidence of iron deficiency anemia, the most common cause of micronutrient-related anemia worldwide [1]. To minimize oxidative and rancidity-based food spoilage, reduce adverse flavor and aroma profile changes, and extend the shelf-life of food products, elemental iron powders are commonly the iron fortificant of choice. However, many commercially available iron powders are relatively untested in vivo [2]. Methods used to extract iron from recycled steel and/or crude mineral ore to produce food-grade, non-heme iron powders vary among manufacturers. The steps during which iron is extracted, processed, and precipitated into relatively pure micro-particulates of elemental iron also change over time due to new technologies [2]. These variations in processing result in physicochemical differences [3] that, as evidence suggests, greatly influence the bioavailability of iron from these sources [4,5]. Hence, more precise information regarding the comparative bioavailability of iron from novel and relatively recently introduced elemental iron powders in the food supply would increase our understanding of the usefulness of such mineral fortificants and advance food enrichment and fortification programs [6].

Based on a review of animal [4,5,6,7,8,9,10,11,12] and human studies [6,13,14,15,16,17,18,19,20,21], a panel of experts convened to review and develop updated micronutrient fortification guidelines found that data on the relative absorption of iron from the types of elemental iron powders presently used to fortify and enrich staple foods, such cereal grain flours, are inadequate [22]. However, for more than four decades, elemental iron powders have been the primary form of iron used by most countries worldwide to fortify and enrich staple foods [23,24]. Earlier studies investigating the nutritional usefulness of elemental iron powders include assessing iron absorption in murine models to determine the hemoglobin regeneration efficiency (HRE) and relative biological value (RBV) using bakery-grade ferrous sulfate monohydrate as a highly bioavailable iron standard [11]; a prior comprehensive review study found excellent agreement between human and rat hemoglobin repletion [12]. As a variety of novel elemental iron powders enter the global food market, it is necessary to thoroughly test the efficacy of these new food fortification agents and determine whether they represent both an efficacious and cost-effective approach to resolve iron deficiency anemia.

The purpose of this study was to determine the HRE and RBV of an electrolytic elemental iron powder compared to bakery-grade ferrous sulfate monohydrate at different concentrations of dietary iron. The aim was to obtain data to better understand the absorption of iron from this type of elemental iron powder and thereby help develop more specific dietary guidelines regarding the use of this form of iron fortificant.

## 2. Materials and Methods

### 2.1. Elemental Iron Powders

This study tested an elemental electrolytic iron powder (EIP) (Industrial Metal Powders (IMP; Maharashtra, India) that meets Food Chemical Codex (FCC) specifications [25]. A “no added iron” diet served as the negative control. Bakery-grade ferrous sulfate monohydrate (FeSO_4_•H_2_O; FS), obtained from a commercial producer (Crown Technologies, Inc., Indianapolis, IN, USA), is an iron salt known for its highly absorbable non-heme iron and was used as a positive control and when determining comparative relative iron bioavailability (RBV) of EIP. EIP and FS were kept in a desiccator under vacuum at room temperature (72 °F) until use. EIP is considered an “elemental iron powder” based on its final chemical form; a relatively pure (99.6% iron *w*/*w*; zero oxidation state) particle of iron with >95% of the particle sizes < 45 µm in diameter. More than 97% of all EIP particles pass through a +325 mesh sieve. The apparent density (g/cm^3^) of EIP is approximately 2.0.

### 2.2. Study Design and Dietary Treatments

An initial batch of 220 weanling male Sprague–Dawley rats (Charles River/SASCO, Wilmington, MA, USA) were used. Rats were individually housed in stainless steel wire-bottom mesh cages in a room controlled for temperature (21 ± 1 °C) and humidity, on a 12 h light:dark cycle. After 24 d of depletion with an iron-deficient diet (1.6 mg iron/kg AIN-93G[M] diet; approximately 1.4 mg iron/kg diet—analyzed iron content), iron-deficient rats, with hemoglobin values between 3 and 6 g/dL (mean ± SEM of 4.1 ± 0.4 g/dL; range 3.1–5.9 g/dL), were then randomly assigned, blocking on hemoglobin, to one of nine different iron repletion period diet groups. Rats consumed repletion diets for 14 d, fortified with either EIP or FS (at 12, 24, 36 mg iron/kg diet), or no added iron; n = 9–12/group. During the iron repletion period, both daily and total food consumption measurements were performed, including adjustments for spilled food. All diets and distilled, deionized water were fed ad libitum. The HRE ratio and, thus, RBV calculations account for body weight and iron intake. All animal procedures followed the Institutional Animal Care and Use Committee (IACUC) procedures at Case Western Reserve University (CWRU), in accordance with NIH guidelines.

EIP and FS were incorporated into diets modified to have a very low base iron content, using vitamin-free casein (Harlan Teklad, Madison, WI, USA) and a high purity cellulose fiber source (Alphacel™; ICN Biomedicals, Irvine, CA, USA). The diets were also phytate-free, with a neutral pH (7.0). The base modified diet [26] (AIN-93G*[M]) composition from which treatment (repletion) period diets were prepared is shown in Table 1. Without added iron, the diet contained approximately 1.4 mg iron/kg diet by analysis. EIP and FS were added into diets taking into account the baseline amount (analyzed) already present in the control (no added iron) group. The modified mineral mix omitted ferric citrate (Harlan Teklad, Madison, WI, USA).

Mixing of EIP and FS into repletion period diets was performed as previously described [27]. A small portion of each diet was taken for analysis to confirm the iron content as previously described [28].

### 2.3. Hemoglobin and Hemoglobin Iron Determinations

Hemoglobin and hemoglobin iron determinations, phlebotomy, and, following repletion, animal anesthetization and sacrifice were performed as previously described [27].

Briefly, the following calculation was used to determine hemoglobin (Hb) iron:

Hb Fe (mg) = BW (kg) × 0.067 × Grams Hb per mL × 3.35 mg Fe

Note: the calculation assumes blood is 6.7% of the body weight (BW; kg) and the hemoglobin iron content is 3.35 mg/g [8,9]. Therefore, hemoglobin iron was determined on the basis of 3.35 mg iron/g hemoglobin and 0.075 L blood/kg body weight [29,30].

### 2.4. Hemoglobin Regeneration Efficiency and Relative Iron Bioavailability

The hemoglobin regeneration efficiency (HRE) of EIP and FS, and the relative biological value (or relative iron bioavailability; RBV) of EIP were determined as described previously [27].

Briefly, HRE and RBV determinations were made at the same concentration of dietary iron (diet-matched), using the analyzed value of iron for each diet. For RBV determinations, bakery-grade ferrous sulfate monohydrate (FeSO_4_•H_2_O; FS) was used as the reference standard (positive control) because of its well-known, high iron bioavailability and the prevalence of data in the literature [5,6].

HRE and RBV were calculated based on the following formulas:

HRE ratio = [Final Hb Fe (mg) − Initial Hb Fe (mg)]/Fe intake (mg total consumed; analyzed diet value)

RBV = Percentage (%) HRE relative to ferrous sulfate monohydrate (FS): HRE ratio of electrolytic iron powder/HRE ratio of the FS group × 100 (diet-matched)

### 2.5. Statistical Analyses

Power analysis was performed based on previous published data [27]. Statistical analyses, including for hemoglobin repletion data and other blood indices, were conducted as previously described [27,28,29,30,31,32].

Briefly, FS served as the standard reference (positive control) for use in RBV determinations. The “no added iron” group served as the negative control (blank), as previously described [27,28]. Differences between HRE and RBV means were tested using Duncan post hoc testing and Tukey’s multiple comparison test using the statistical package SAS (SAS Version 10.2, SAS Institute, Cary, NC, USA). Significance was set at *p* ≤ 0.05. Values are expressed as mean ± SEM. Charts and graphical illustration of data and results were performed using GraphPad Prism (Software version 10.2; GraphPad, Boston, MA, USA).

## 3. Results

### 3.1. Hemoglobin and Hemoglobin Iron Change

**Figure 1:**
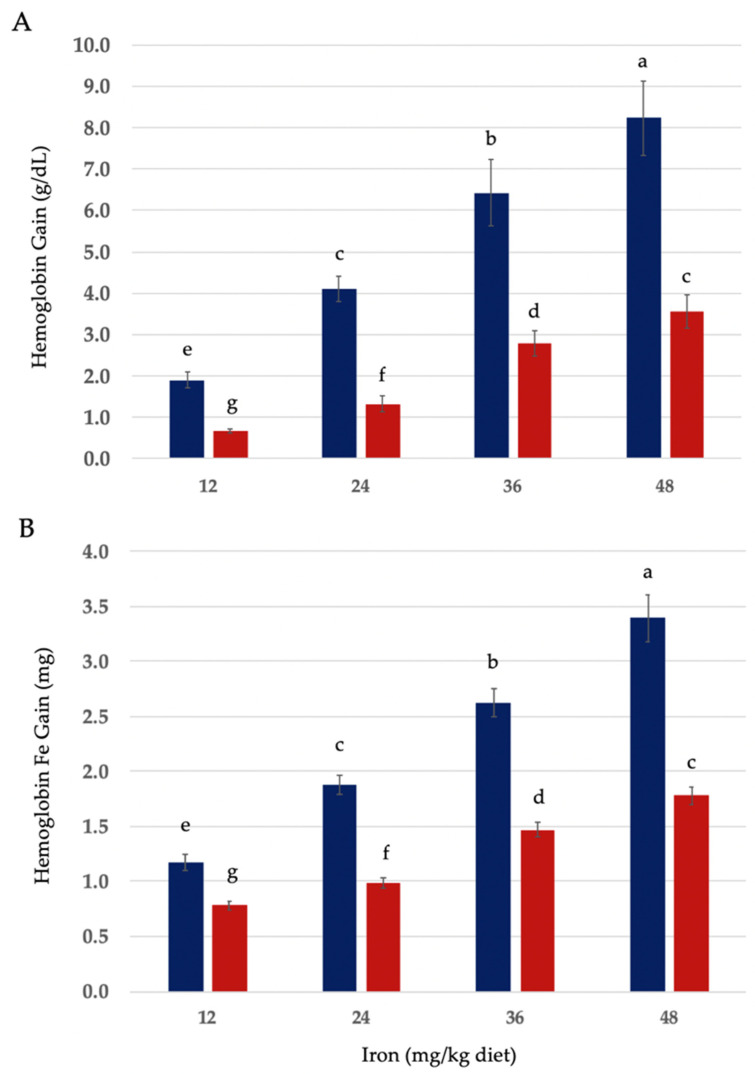
(**A**) Hemoglobin (Hb) gain (g/dL) and (**B**) hemoglobin iron (Fe) gain in anemic rats fed graded quantities of ferrous sulfate monohydrate (FeSO_4_•H_2_O) or electrolytic elemental iron powder for a 14-day repletion period (blue and red bars, respectively). Values are mean ± SEM (n = 9–12/group). Different letters are used to denote significant differences (*p* ≤ 0.05), from higher to lower hemoglobin and hemoglobin Fe gain. Hb Fe (mg) = BW (body weight; kg) × 0.067 × Grams Hb per mL × 3.35 mg Fe.

Hematological values of anemic rats fed no added iron, electrolytic elemental iron powder (EIP), or bakery-grade ferrous sulfate monohydrate (FS) are shown in Table 2. Food intake and weight gain were positively associated with increasing dietary iron in all treatment groups (data in Table 2). Iron intake (mg/day) was positively associated with dietary iron concentration (Figure S1). Although hemoglobin change and hemoglobin iron (Fe) gain in anemic rats fed EIP or FS were positively associated with iron concentration in the diet, both of these indices were greater at each level of dietary iron for FS (Figure 1A,B).

### 3.2. Hemoglobin Regeneration Efficiency and Relative Iron Bioavailability of Electrolytic Elemental Iron Powder

Hemoglobin regeneration efficiency (HRE) ratios of EIP and FS, and relative iron bioavailability (RBV) of EIP as compared to FS are shown in Table 3. For EIP, the HRE ratios of diets at 12, 24, 36, and 48 mg iron/kg diet were 0.356 ± 0.04, 0.205 ± 0.02, 0.197 ± 0.01, and 0.163 ± 0.008, respectively. For FS, the HRE ratios of diets at 12, 24, 36, and 48 mg iron/kg diet were 0.552 ± 0.06, 0.389 ± 0.04, 0.347 ± 0.05, and 0.307 ± 0.03, respectively. The RBV (%) of iron from EIP in diets at 12, 24, 36, and 48 mg iron/kg diet were 64.5 ± 3.7, 59.1 ± 3.9, 50.6 ± 2.8, and 54.3 ± 3.3, respectively. HRE ratios and RBV values that significantly differed (*p* > 0.05), as well as mean ± SEM for all diet groups are shown in Table 3.

**Figure 2:**
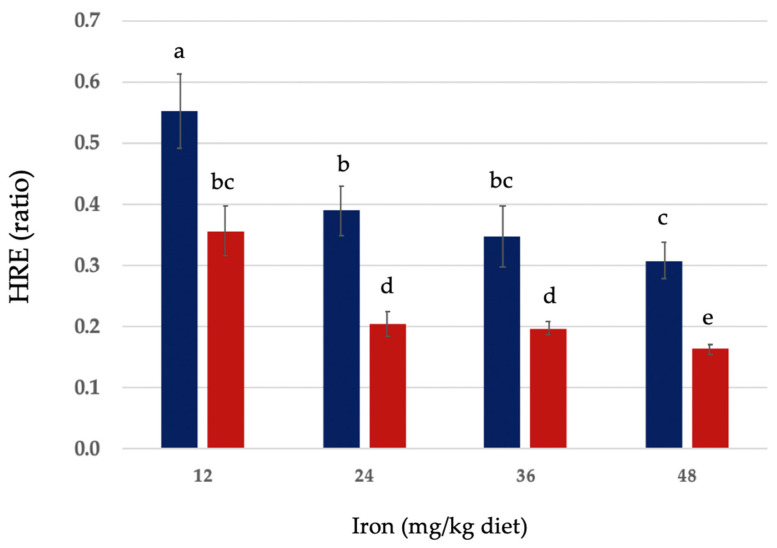
Hemoglobin (Hb) regeneration efficiency (HRE) in anemic rats fed graded quantities of ferrous sulfate monohydrate (FeSO_4_•H_2_O) and electrolytic elemental iron powder for a 14-day repletion period (blue and red bars, respectively). Values are mean ± SEM (n = 9–12/group). Different letters are used to denote significant differences (*p* ≤ 0.05), from higher to lower HRE. HRE ratio = [Final Hb Fe (mg) − Initial Hb Fe (mg)]/Fe intake (mg total consumed).

**Figure 3:**
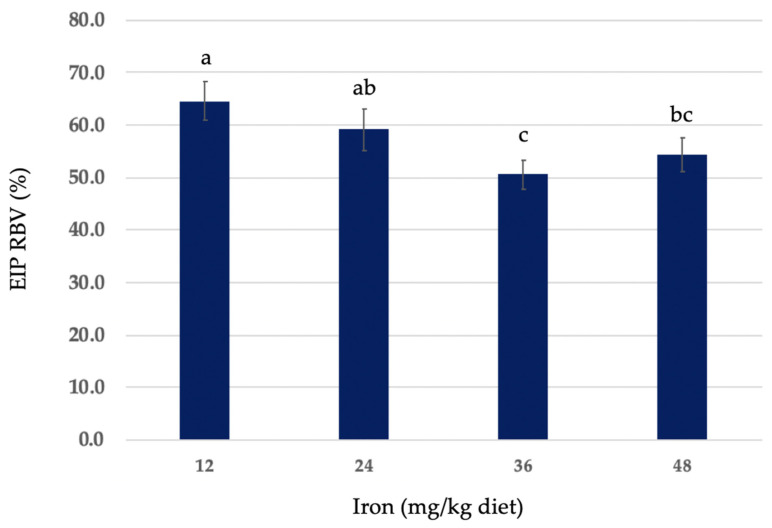
Relative iron bioavailability (RBV) of electrolytic elemental iron powder (EIP) vs. ferrous sulfate monohydrate (FeSO_4_•H_2_O) at different concentrations of dietary iron. Values are mean ± SEM (n = 9–12/group). Different letters are used to denote significant differences (*p* ≤ 0.05), from higher to lower RBV. RBV = percentage (%) HRE relative to ferrous sulfate monohydrate (FS): HRE ratio of EIP/HRE ratio of the FS group × 100 (diet-matched).

The HRE ratio calculation accounts for iron intake. HRE rations of EIP and FS at each level of dietary iron are shown in Figure 2. RBVs of EIP at each level of dietary iron (iron concentration in the diet matched to FS) are shown in Figure 3.

## 4. Discussion

The rat hemoglobin repletion assay has been found to be both a valid model and a proficient approach to studying the abilities of different types of elemental iron fortificants to correct iron deficiency anemia [6,27,28,29,30,31,32,33,34,35,36,37,38]. Findings from this study show that at the concentrations of iron tested, this relatively novel electrolytic elemental iron powder (EIP) is a useful fortification agent for replenishing hemoglobin and correcting iron deficiency anemia. Additionally, our data are unique in illustrating the comparative HRE ratios and RBVs of EIP vs. bakery-grade ferrous sulfate monohydrate (FS) at four different concentrations of iron in the diet.

Our data show that hemoglobin change and hemoglobin iron gain in anemic rats fed EIP or FS were positively associated with iron concentration in the diet and that both of these indices were greater at each level of dietary iron for FS. These findings (positive associations between dietary iron, hemoglobin and hemoglobin iron change) are in agreement with studies that have investigated the hematological effects of other forms of elemental iron powders [6,7,9,11,27,28,33,34,35,36]. Because of the known higher bioavailability of iron from FS [22,27,28], its use as a positive control in our study allowed for direct comparisons between EIP and FS at each level of the dietary iron. Through these direct, diet-matched comparisons, we also found that HRE ratios of FS were significantly higher (*p* ≤ 0.05) than EIP’s at each level of dietary iron. Our data concur with the findings of previous studies of elemental iron powders in this regard, when compared to FS or other iron salts [12,27,28,39,40,41,42,43,44].

The RBV of iron from EIP ranged from approximately 50–65% that of FS, with the 36 mg iron/kg diet level RBV being the lowest, albeit not significantly different (*p* > 0.05) from the 48 mg iron/kg diet group. However, our findings are overall in line with similar studies that have tested a range of iron concentrations in the diets of rats and humans and the effect on hemoglobin repletion [27,28,36,38,42]. The range of RBVs we found in this study for EIP at the dietary concentrations of iron tested, especially at 12 mg Fe/kg diet, are within approximately 10–15% of the RBVs for other types of electrolytic iron powders [41]. Other studies using murine models, testing a variety of forms of elemental iron powders, found that CO-reduced, H-reduced, and electrolytic iron powders, in comparison to FS, yielded RBVs of 32%, 25%, and 70%, respectively [44,45]. Overall, our data for EIP concur with the findings compiled in a previous meta-analysis [2] of animal and human studies, and a task force assigned to review international fortification approaches, which studied the usefulness of elemental iron for cereal flour fortification [46].

One limitation of this study may be that additional (more than four) concentrations of dietary iron were not tested. However, this study was designed to test a range of diet iron concentrations that approximate human daily iron intake when consuming a variety of iron-fortified foods [47]. Therefore, data from this study may be useful when considering new food fortification policies and guidelines regarding elemental iron powders, as other micronutrient fortification programs have suggested [48]. 

Overall, data from this study show that EIP is efficacious when compared to FS to increase hemoglobin and resolve iron deficiency anemia. This finding is reinforced when comparing hemoglobin and hemoglobin iron gain values, which, for the control, were lower than 0, representing how in the control group these indices became slightly lower during the course of the repletion period. This was not surprising because the control had no added iron and was used for comparison to the iron fortified diets. Additionally, our findings concur with a prior study [27], which revealed that an effective RBV for electrolytic elemental iron powders, such as EIP, may be attained at relatively lower, rather than higher, concentrations of iron in the diet; a key finding of this study was that for both EIP and FS, HRE ratios were inversely associated with increasing dietary iron. This inverse association may indicate that negative feedback is taking place. In future studies, determining the influence of EIP, and FS, on the regulatory hormone, hepdicin and the erythroid regulator, erythroferrone would be beneficial in this regard. Further, it would also be beneficial in future studies to determine the relative influence of EIP and FS on body iron concentration and distribution (e.g., serum iron, total iron binding capacity and transferrin saturation, liver iron, adipocyte iron and reticuloendothelial iron).

Because current iron fortification guidelines regarding elemental iron powders worldwide commonly suggest incorporation of approximately twice the concentration of some types of elemental iron powders based on their comparative (to FS) 50% RBV, our findings may assist in the development of more proficient and economical iron fortification approaches using elemental iron powders. Our findings also support the use of EIP in fortified and enriched foods and in iron and/or multimineral supplements in clinical settings in near-equivalent dietary iron concentrations to iron salts.

Due to the vital importance of treating iron deficiency anemia globally [49], it is recommended that further studies, including human clinical trials, be undertaken to determine what an optimal dietary level of EIP may be under a variety of food manufacturing and preparation conditions, and in the presence of dietary enhancers and inhibitors of non-heme iron absorption in order to optimize non-heme iron bioavailability.

## 5. Conclusions

In sum, this electrolytic elemental iron powder is useful as a food fortification agent to replenish hemoglobin and correct iron deficiency anemia. Taking into account the lower cost of this iron powder and its propensity to minimize unfavorable organoleptic changes in foods during storage vs. ferrous sulfate and other iron salts, the inclusion of this form of iron powder as part of fortification or enrichment strategies in a variety of foods may be both advantageous and efficacious.

## Figures and Tables

**Table 1:** Baseline diet composition ^1^, from which treatment diets were prepared by adding iron (Fe; as electrolytic elemental iron powder or bakery-grade ferrous sulfate monohydrate).

Formula		g/kg
Casein, low Cu and Fe		200.0
Sucrose		314.5
Corn starch		315.0
Soybean oil		70.0
Cellulose, microcrystalline (Alphacel™)		50.0
Mineral Mix modified, no added iron (81062) ^2^		35.0
Vitamin Mix, AIN-93-VX (40077) ^3^		10.0
L-cysteine		3.0
Choline bitartrate		2.5
TBHQ, antioxidant ^4^		0.014
Macronutrient	% dry weight	% kcal
Protein	17.7	17.8
Carbohydrate	69.8	70.4
Fat	5.2	11.8

^1^ Ref: [26]. The base AIN-93G[M] diet was prepared by Harlan Teklad (Catalog #TD.99397; Harlan Teklad, Madison, WI, USA). ^2^ Calcium at 3.5 g/kg diet. ^3^ Ascorbic acid at 200 mg/kg diet. ^4^ Tertiary-butylhydroquinone (TBHQ). Catalog numbers are also shown for mineral and vitamin mixes. All diet ingredients were obtained from Harlan Teklad, Madison, WI, USA.

**Table 2:** Food and iron (Fe) intake and growth, and hemoglobin iron (Fe) and change in anemic rats fed graded quantities of the electrolytic elemental iron powder (EIP) or ferrous sulfate monohydrate (FS) for a 14-day repletion period ^1,2^.

	Control (No Added Iron)	Electrolytic Iron Powder (EIP)				Ferrous Sulfate (FS)			
Diet Code	C	EIP-1	EIP-2	EIP-3	EIP-4	FS-1	FS-2	FS-3	FS-4
Diet Fe (mg/kg) Calculated (Analyzed)	1.6 (1.4)	12 (11.7)	24 (24.8)	36 (35.6)	48 (48.1)	12 (11.8)	24 (24.2)	36 (36.3)	48 (46.9)
Food intake (g/day)	11.7 ± 0.61 ^d^	13.4 ± 0.66 ^c^	13.8 ± 0.69 ^bc^	15.0 ± 0.84 ^ab^	16.2 ± 0.91 ^ab^	12.9 ± 0.54 ^c^	14.2 ± 0.75 ^b^	14.9 ± 0.81 ^b^	16.8 ± 0.86 ^a^
Fe intake (mg/day)	0.016 ± 8^−4 e^	0.157 ± 6^−3 d^	0.342 ± 0.04 ^c^	0.534 ± 0.05 ^b^	0.779 ± 0.08 ^a^	0.152 ± 7^−3 d^	0.344 ± 0.02 ^c^	0.541 ± 0.06 ^b^	0.788 ± 0.09 ^a^
Body weight (g) Initial (Gain)	83.9 ± 3.7 ^a^ (15.2 ± 0.8 ^c^)	84.4 ± 3.7 ^a^ (53.4 ± 2.8 ^ab^)	83.2 ± 3.3 ^a^ (54.5 ± 3.1 ^ab^)	83.1 ± 3.7 ^a^ (56.1 ± 3.5 ^ab^)	84.2 ± 3.9 ^a^ (58.7 ± 3.8 ^ab^)	84.7 ± 3.2 ^a^ (53.8 ± 2.9 ^ab^)	84.2 ± 3.3 ^a^ (56.1 ± 2.4 ^ab^)	83.1 ± 3.7 ^a^ (57.5 ± 3.1 ^ab^)	85.2 ± 3.6 ^a^ (62.4 ± 3.7 ^a^)
Hemoglobin (g/dL) Initial (Gain)	4.63 ± 0.4 ^a^ (−0.42 ± 0.03 ^h^)	4.81 ± 0.5 ^a^ (0.66 ± 0.05 ^g^)	4.70 ± 0.3 ^a^ (1.32 ± 0.2 ^f^)	4.75 ± 0.4 ^a^ (2.79 ± 0.3 ^d^)	4.84 ± 0.7 ^a^ (3.56 ± 0.4 ^c^)	4.83 ± 0.3 ^a^ (1.9 ± 0.2 ^e^)	4.62 ± 0.2 ^a^ (4.11 ± 0.3 ^c^)	4.65 ± 0.4 ^a^ (6.42 ± 0.8 ^b^)	4.74 ± 0.6 ^a^ (8.23 ± 0.9 ^a^)
Hemoglobin Fe ^3^ Gain (mg)	−0.064 ± 6^−3 h^	0.781 ± 0.04 ^g^	0.983 ± 0.05 ^f^	1.469 ± 0.07 ^d^	1.779 ± 0.08 ^c^	1.174 ± 0.07 ^e^	1.876 ± 0.09 ^c^	2.626 ± 0.13 ^b^	3.390 ± 0.21 ^a^

^1^ Values are mean ± SEM (n = 9–12/group). ^2^ Means in same row of same diet type that do not share the same letter(s) are significantly different (*p* ≤ 0.05). ^3^ Hb Fe (mg) = BW (body weight; kg) × 0.067 × Grams Hb per mL × 3.35 mg Fe.

**Table 3:** Hemoglobin regeneration efficiency (HRE) and relative iron bioavailability (RBV) in anemic rats fed graded quantities of the electrolytic elemental iron powder (EIP) or ferrous sulfate monohydrate (FS) for a 14-day repletion period ^1,2^.

	Electrolytic Iron Powder (EIP)				Ferrous Sulfate (FS)			
Diet Code	EIP-1	EIP-2	EIP-3	EIP-4	FS-1	FS-2	FS-3	FS-4
Diet Fe (mg/kg) Calculated (Analyzed)	12 (11.7)	24 (24.8)	36 (35.6)	48 (48.1)	12 (11.8)	24 (24.2)	36 (36.3)	48 (46.9)
HRE ratio ^3^	0.356 ± 0.04 ^bc^	0.205 ± 0.02 ^d^	0.197 ± 0.01 ^d^	0.163 ± 8^−3 e^	0.552 ± 0.06 ^a^	0.389 ± 0.04 ^b^	0.347 ± 0.05 ^bc^	0.307 ± 0.03 ^c^
RBV ^4^	64.5 ± 3.7 ^a^	59.1 ± 3.9 ^ab^	50.6 ± 2.8 ^c^	54.3 ± 3.3 ^bc^	-	-	-	-

^1^ Values are mean ± SEM (n = 9–12/group). ^2^ Means in same row that do not share same letter(s) are significantly different (*p* ≤ 0.05). ^3^ HRE ratio = [Final Hb Fe (mg) − Initial Hb Fe (mg)]/Fe intake (mg total consumed). ^4^ RBV = Percentage (%) HRE relative to ferrous sulfate monohydrate (FS): HRE ratio of EIP/HRE ratio of the FS group × 100 (diet-matched).

## Data Availability

The original contributions and data on which findings are presented in this study are included within the article and Supporting Material that accompany this submission.

## Supplementary Materials

The following supporting information can be downloaded at https://www.mdpi.com/article/10.3390/nu16172833/s1: Figure S1: Iron (Fe) intake (mg/day) of anemic rats fed graded quantities of electrolytic elemental iron powder or ferrous sulfate monohydrate (FeSO_4_•H_2_O) for a 14-day repletion period (red and blue bars, respectively). Values are mean ± SEM (n = 9–12/group). Different letters are used to denote significant differences (*p* ≤ 0.05), from higher to lower iron intake.
