# Supplementary material for: An Electrolytic Elemental Iron Powder Effectively Regenerates Hemoglobin in Anemic Rats and Is Relatively Well Absorbed When Compared to Ferrous Sulfate Monohydrate
Source: Nutrients. 2024 Aug 24;16(17):2833. doi: 10.3390/nu16172833 (PMC11397326; doi:10.3390/nu16172833)
Supplement: Supplementary file 1 [file nutrients-16-02833-s001.zip › nutrients-3163472-supplementary.pdf]

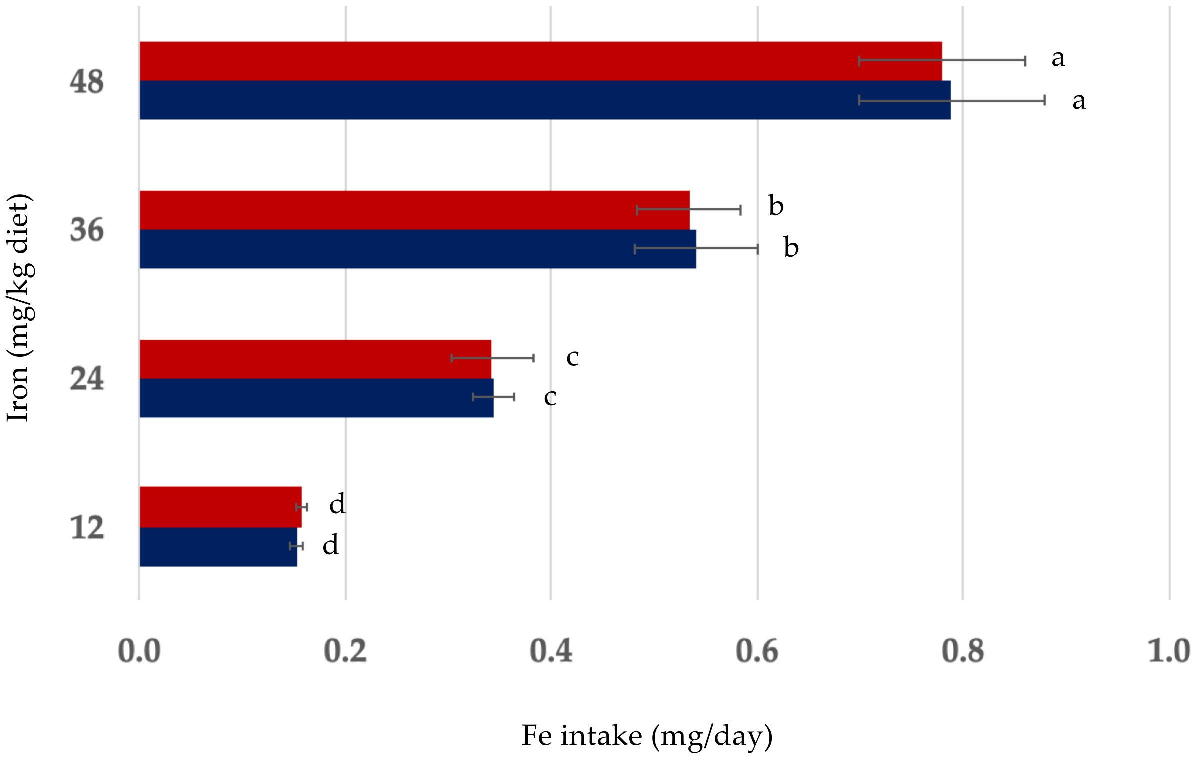

Fig. 1S. Iron (Fe) intake (mg/day) of anemic rats fed graded quantities of electrolytic elemental iron powder or ferrous sulfate monohydrate ($FeSO_4 \bullet H_2O$) for a 14-day repletion period (red and blue bars, respectively). Values are mean ± SEM (n=9-12/group). Different letters are used to denote significant differences ($p \leq 0.05$), from higher to lower iron intake.
